# The effects of male peers on the educational outcomes of female college students in STEM: Experimental evidence from partnerships in Chemistry courses

**DOI:** 10.1371/journal.pone.0235383

**Published:** 2020-07-09

**Authors:** Robert Fairlie, Glenn Millhauser, Daniel Oliver, Randa Roland

**Affiliations:** 1 Department of Economics, University of California, Santa Cruz, California, United States of America; 2 Department of Chemistry, University of California, Santa Cruz, California, United States of America; 3 Education Research Alliance, Tulane University, New Orleans, Louisiana, United States of America; Universitá degli Studi di Bergamo, ITALY

## Abstract

A major concern among universities around the world is that female students face gender bias, discrimination and related barriers in male-dominated STEM fields. To investigate this concern, we conducted a novel large-scale experiment of interactions between female and male students in one of the most important gateway courses for the Sciences and a course in which students interact one-on-one extensively throughout the term. Over the past four years, at a large public research university, we randomly paired every student enrolled in an introductory Chemistry lab (3,902 students and total N = 5,537). Using precise estimates from the experiment, we provide novel evidence that female students are not negatively affected academically by male partners. When assigned a male partner, female students do not receive lower scores or grades, and they are no more likely to drop the course or not continue in Chemistry or a STEM field. We also find that academically weaker female students are not negatively affected by male students and that female students are not negatively affected when paired with academically stronger male students. Although previous studies have documented that female students self-report experiencing gender bias from male peers in STEM, importantly, we do not find evidence that female students are negatively affected by male peers in intensive, long-term pairwise interactions in their course grades or future STEM course taking. The findings provide hopeful news for future trends in female representation in STEM fields.

## Introduction

The underrepresentation of women in STEM fields is one of the most pressing problems in higher education. The disparity exists around the world and contributes substantially to gender earnings inequality because STEM jobs are typically high paying [1–4]. Of particular concern is that the lack of role models, stereotype threat, gender bias, and fear of competition contribute to fewer women taking courses and graduating in STEM fields [5,6,7,8,9]. These negative influences may be heightened when female students interact frequently with male students in male-dominated STEM fields. Female college students in STEM report high levels of gender bias from male peers [10], and STEM-related gender bias from classmates is negatively related to STEM motivation and career aspirations [11]. Female students also often report leaving STEM because of a negative climate characterized by intense competition, lack of support and discouraging peers [12], and female students are negatively affected by competitive environments in STEM classes whereas male students are not affected [13]. All of these factors may lead to frequent and intensive male-female student interactions having a negative effect on female students in STEM.

To investigate these concerns, we conducted a large-scale experiment of interactions between female and male students in an essential, gateway STEM course and one in which students work naturally as partners—first-year Chemistry laboratory classes. To our knowledge, it is the first experimental study to test directly whether female students are negatively affected in course performance and STEM continuation when interacting one-on-one with male peers in the classroom. We focus on course grades and future course taking as opposed to self-reports of gender bias to identify effects on measurable academic outcomes that count towards graduating with a degree in a STEM field.

General chemistry laboratories provide an ideal and well-controlled environment for assessing gender interactions. Our selection of these laboratory courses is based on the following four criteria. First, we sought an environment in which students work closely together in pairs throughout the term but are graded independently on all tests and assignments. This is not achievable in large lecture halls where students choose their seating and interaction space. Second, lab courses are important—they are an essential, gateway course for the Sciences and many other STEM majors. This criterion ensures that we are evaluating a cross-section of science students. Third, we wanted a course that engages a broad set of qualitative and quantitative skills including scientific techniques, mathematical modeling and statistics. Fourth, we required courses with partner assignment to generate close interactions and allow for random matching of students. By meeting these four criteria, chemistry labs allow us to evaluate quantitatively whether female students experience negative interaction effects from male students, as reflected in performance and subsequent course taking, early in their in their STEM college experience—a juncture at which they are especially vulnerable to leaving a STEM major [12].

Many factors underlie why female students leave the sciences and other STEM fields [13,14], and the process is complicated and spans many years [15,16]. The introductory sequence in Chemistry certainly does not constitute the only barrier in this process but it is likely to represent an important one. Focusing on Chemistry labs in particular may generate some insights into factors related to gender bias from male peers.

For the experiment, students in every lab section associated with the first-year sequence in Chemistry at a large public research university were randomly assigned a partner. The study involves 3,902 students over four academic years (total N = 5,537). The experiment specifically tests whether the academic outcomes of female college students are negatively affected by being paired with male students, and whether any negative effects depend on the student’s ability, partner’s ability, and whether they have a female graduate student teaching assistant. For example, low ability female students might be more negatively affected by male peers than high ability female students. We also determined downstream effects of various pairings by examining whether pairing with male students inhibits continuation to more advanced chemistry classes or ultimate selection of a STEM major. Randomly assigning students to lab partnerships allows us to avoid the common and serious estimation bias resulting from self-selection (i.e. students might choose to work with their friends, other students like themselves, or students who can help them the most).

## Methods

### Protocol approval

The study received joint approval from the Chemistry and Economics Departments at the University of California, Santa Cruz. The project received University of California Human Subjects approval by the Office of Research Compliance Administration. Our protocol was approved as exempt from IRB review under Category 1, which covers research conducted in commonly accepted educational settings involving normal education practices.

### Experimental setting

At a large, public university, we randomized all student pairings in introductory Chemistry labs from Winter Quarter 2015 to Spring Quarter 2018. The University has a total enrollment of roughly 20,000 students. Total enrollment in all labs observed for our study is 5,537 (3,902 students). Enrollment in the 330 unique Chemistry labs is capped at 18 (mean = 16.8). Average enrollment in the large-lecture introductory Chemistry courses is 348.

Chemistry labs provide an important setting in which to study gender interactions because students work in pairs that are assigned for the entire term. In a classroom of 300 students, or even 30 students, it is very difficult to identify which classmates have the most influence on a particular student and students can generally choose who they sit near and interact with in the class potentially avoiding or reducing gender bias and discrimination from peers. In Chemistry labs, students work very closely together but take individual assessments and are graded on their own knowledge of the subject material. The one-to-one matching in Chemistry labs removes this measurement problem and provides an intensive interaction between students. Additionally, we avoid the concern with previous work that random assignment to classrooms creates little, or essentially no, variation in female shares of classrooms when there are large classes. The assigned lab partner is either male (female share = 0) or female (female share = 1) instead of variation that might range from female share = 30 percent to 40 percent, for example.

The Introduction to Chemistry sequence at the university covers a standard set of topics, similar to other large research universities. The laboratory classes associated with this sequence are also standard. The sequence requires a minimum of pre-calculus before enrolling, but most students have already taken calculus. The sequence involves extensive use of math throughout the coursework. Students generally take Chem 1A, 1B and 1C in consecutive quarters. The two labs (Chem 1M and 1N) are associated with the second and third quarter courses in the sequence, respectively. Chem 1M requires Chem 1B as a prerequisite or with concurrent enrollment; the same holds for Chem 1N and Chem 1C. However, these laboratory courses are not interdependent on each other and may be taken in either order. With regard to topics, Chem 1M emphasizes analytical techniques, such as determination of empirical formulas, along with chemical kinetics and introductory spectroscopy. Chem 1N emphasizes chemical thermodynamics, acids and bases, solubility and electrochemistry. The experiments are similar to those traditionally found in other introductory chemistry series and are designed to emphasize concepts covered in the Chem 1B and 1C lectures. Although Chem 1M is not a prerequisite for Chem 1N, the majority of students nevertheless take them in that order. Moreover, most students take both laboratories.

The Introduction to Chemistry sequence which includes the labs is the gateway requirement to a diverse set of STEM majors, including Chemistry, Biology, Bioengineering, Environmental Studies, Environmental Science, Earth Sciences, Ecology and Neuroscience. It is also commonly taken by students in many other STEM majors (e.g. Physics, Computer Science, and Cognitive Science). Chemistry labs develop a broad skillset including a strong mathematical component (e.g. statistics, linear regression, physical processes, experimental measurement, and instrumentation).

The laboratory curriculum, physical equipment and space are standardized across sections. The laboratory equipment and materials are uniformly disbursed from a central laboratories manager. Lab sections are held in eight different laboratory classrooms along one hallway in the Chemistry instruction building. The standardization across lab sections provides one of the most controlled environments for studying social interactions between students possible on a college campus.

At both the undergraduate and graduate level, women are underrepresented in Chemistry, which is similar to most other STEM fields. Nationally, women receive 48 percent of Chemistry bachelor’s degrees compared to 57 percent of all bachelor’s degrees [17]. At the university 43 percent of Chemistry majors are female (S1 Table in S1 Appendix). The Chemistry labs at UCSC generally reflect the diverse UCSC student population by race and ethnicity. On the other hand, the labs have a larger female representation than the overall representation in all courses and majors in Chemistry. We also compare our results to those for all U.S. students receiving degrees in Chemistry or STEM. Our lab students have a higher percentage of women compared to the U.S. total, but this is likely due to Chemistry labs enrolling students from other fields such as Biology. A comparison of the percentage of UCSC students majoring in Chemistry to the national average looks more similar for female percentage. Certainly, UCSC is a more ethnically and racially diverse campus than the national average, but perhaps predicts future trends at colleges.

Turning to higher levels of education, women also receive only 37 percent of doctorates in Chemistry and this percentage has remained unchanged over the past decade [18]. At the university, 38 percent of graduate students in Chemistry are female. Only 20 percent of faculty in Chemistry are female.

### Statistical model

To explore the effects of female students interacting with male students in Chemistry lab pairings we estimate the following equation.

$$Y_{i}=\beta_{1}X_{i}+\beta_{2}F_{i}+\beta_{3}F_{i}*M_{i}{}^{PT}+\beta_{4}M_{i}*F_{i}{}^{PT}+\gamma_{s}+\varepsilon_{is},$$ (1)

where Y_i_ is the student’s academic outcome, X_i_ is a vector of background characteristics of the student, F_i_ = 1 if the student is female, M_i_ = 1 if the student is male, F_i_^PT^ = 1 if the student’s partner is female, M_i_^PT^ = 1 if the student’s partner is male, γ_s_ are unique lab section fixed effects, and ε_is_ is the error term. We include the female indicator variable, F_i_, to control for underlying female/male differences in academic outcomes. β_3_ captures the effect of female students being partnered with male students relative to being partnered with female students. The coefficient estimate will be negative if female students perform worse when matched with male lab partners. The coefficient can be interpreted in the context of a field experiment in which for female students the treatment condition is being assigned a male partner and the control condition is being assigned a female partner. β_4_ captures whether male students are affected by being partnered with a female student relative to being partnered with a male student. A comparison of β_3_ and β_4_ provides evidence on whether there is symmetry in gender interaction effects.

We estimate Eq (1) for four primary academic outcomes. First, we measure overall course performance using the numeric continuous score (i.e. scale of 0–100). Final scores in the class are based on the following assignments: Written procedure and data tables (7 assignments) 25%; Online prelabs (7) 5%; Online in-lab assignments (7) 35%; Online reviews (7) 5%; Formal abstracts (2) 10%; Online quizzes (7) 10%; Scholarship and week 1 worksheet 10%. We rescale this score by demeaning and dividing by the standard deviation. Second, we measure performance using the letter grade in the course converted to a 4-point scale (i.e. scaled similarly as a GPA measure, 0–4.3). Third, we measure performance using whether the student passed the course. Fourth, we measure performance using whether the student dropped the course. Eq (1) is estimated using ordinary least squares (OLS) for all four outcome measures. We find similar estimates for marginal effects when estimating Probit or Logit models for the two indicator outcomes, passing the course and dropping the course.

A key control included in all regressions is the student’s grade in Chemistry 1A, which is the first lecture course taken in the introductory sequence. Chemistry 1A is taken in a prior term to enrollment in the labs. To control more thoroughly for differences across student abilities in Chemistry we include the full set of dummy variables for letter grades. As expected, grades in Chemistry 1A are a very strong predictor of performance in the lab. We also use grades in this course to define low and high ability students and lab partners in later analyses (i.e. Tables 1 and 2). Alternatively distinguishing between low and high ability based on a student’s performance in all previous courses (i.e. prior GPA) provides similar results (S3 and S4 Tables in S1 Appendix and report these alternative estimates).

**Table 1:** Regression coefficients for main outcomes. Linear regressions control for baseline lab sections, full grade distribution in Chem 1A prior to labs, ethnicity, gender, Educational Opportunity Programs status, year in college, major interest, and declaration of major. Standard errors (in parentheses) are clustered by lab sections.

		Numeric score	Grade (4 point scale)	Passed course	Dropped course
		1	2	3	4
Female student with		0.0038	-0.0099	0.0052	-0.0046
	male partner	(0.0338)	(0.0154)	(0.0083)	(0.0083)
Male student with		0.0344	0.0021	0.0075	-0.0036
	female partner	(0.0520)	(0.0236)	(0.0106)	(0.0101)
Observations		4,968	4,976	5,246	5,246
R-squared		0.2844	0.1807	0.1073	0.1058
Mean (Dep. var.)		0.0000	3.8219	0.9476	0.0499
SD (Dep. var.)		1.0000	0.4438	0.2229	0.2178

*** p<0.01, ** p<0.05, * p<0.1.

**Table 2:** Regression coefficients for main outcomes by ability of student. Linear regressions control for baseline lab sections, full grade distribution in Chem 1A prior to labs, ethnicity, gender, gender by ability, Educational Opportunity Programs status, year in college, major interest, and declaration of major. Standard errors (in parentheses) are clustered by lab sections.

		Numeric score	Grade (4 point scale)	Passed course	Dropped course
		1	2	3	4
Female student of low ability with		0.0568	-0.0039	0.0113	-0.0112
	male partner	(0.0550)	(0.0261)	(0.0138)	(0.0139)
Female student of high ability with		-0.0434	-0.0232	0.0007	-0.0001
	male partner	(0.0370)	(0.0159)	(0.0110)	(0.0109)
Male student of low ability with		0.0136	-0.0084	0.0107	-0.0025
	female partner	(0.1111)	(0.0494)	(0.0199)	(0.0187)
Male student of high ability with		0.0334	-0.0051	0.0055	-0.0064
	female partner	(0.0496)	(0.0215)	(0.0126)	(0.0122)
Observations		4,761	4,769	5,023	5,023
R-squared		0.2848	0.1768	0.1118	0.1108
Mean (Dep. var.)		0.0265	3.8335	0.9490	0.0490
SD (Dep. var.)		0.9724	0.4224	0.2199	0.2158

*** p<0.01, ** p<0.05, * p<0.1.

The ability measures allow us to also explore potential heterogeneity in the results by student ability. For example, low-ability female students might be affected differently by male partners than high-ability female students are affected by male partners. To explore this question we estimate the following equation:

$$Y_{i}=\beta_{1}X_{i}+\beta_{2}F_{i}+\beta_{3}LF_{i}*M_{i}{}^{PT}+\beta_{4}HF_{i}*M_{i}{}^{PT}+\beta_{5}LM_{i}*F_{i}{}^{PT}+\beta_{6}LM_{i}*F_{i}{}^{PT}+\gamma_{s}+\varepsilon_{is},$$ (2)

In this case, we have two estimates for female students and their interactions with male partners. Note that the ability level of the student is controlled for in X_i_. β_3_ captures the effect of low-ability female students being partnered with male students relative to being partnered with female students, and β_4_ captures the effect of high-ability female students being partnered with male students relative to being partnered with female students.

Additional controls included in all regressions are baseline lab section fixed effects, a detailed set of race/ethnicity indicators, Educational Opportunity Programs status, year in college, major interest, and declaration of major. The estimates are robust to excluding controls for student characteristics, which is expected because of the random assignment of male and female partners. It is important to note that the inclusion of lab section fixed effects in Eq (1) controls for the variation in performance due to different instructors, teaching assistants, rooms, lab courses (i.e. Chemistry 1M and 1N), academic years/terms, section times, and days of the week. Importantly, it also implicitly controls for the female/male mix of all students in the lab. The inclusion of lab section fixed effects makes it unnecessary, and in fact mathematically impossible, to include measures of these non-student characteristics in the equation.

### Randomization process and balance check

To study gender interactions in Chemistry labs we randomly assigned partners in all introductory Chemistry lab courses over the past four years. Students were assigned partners on the first day of sections, and these partnerships were maintained for the entire term. The process of randomization was deliberately transparent—students drew folded slips of paper with numbers between 1 and 9 from a large beaker. Students with matching numbers were paired. When only 16 students (or an even amount of students below 18) were present for the draw, pairs of slips with the same number were either omitted from the beaker, or students with the lowest unmatched numbers were matched. When only 17 students (or an odd number of students) were present for the draw, the non-matching student was added to the lowest numbered pair. We drop these observations which represent only 2 percent of all partnerships.

To check statistical validity, we perform a balance check of randomization in our experiment. As expected with random assignment of lab partners we find that students paired with female students are observably similar to students paired with male students. Table 3 reports detailed demographic and academic characteristics of both groups of students and confirms that in all cases there are no statistically significant differences between the two groups.

**Table 3:** Balance in characteristics by treatment. Differences are regression adjusted for lab section fixed effects and classmate composition of specified characteristic.

	Female partner	Male partner	Difference	(P-Value)
Female	0.5804	0.5821	0.0017	(0.5884)
White	0.2939	0.2915	-0.0024	(0.2599)
Asian	0.3144	0.3164	0.002	(0.3374)
Hispanic/Latina(o)	0.2725	0.2734	0.0009	(0.6709)
African-American/Black	0.0206	0.0211	0.0005	(0.4180)
EOP student	0.343	0.3419	-0.0011	(0.6919)
Freshman	0.2453	0.245	-0.0003	(0.8472)
Sophomore	0.5896	0.5878	-0.0018	(0.3784)
Junior	0.1224	0.125	0.0026	(0.1070)
Senior	0.0427	0.0419	-0.0008	(0.3474)
Prior Chem 1A grade	2.8922	2.8911	-0.0011	(0.8041)
Pre-GPA	3.2056	3.2039	-0.0017	(0.4535)

*** p<0.01, ** p<0.05, * p<0.1.

## Results

We turn to the estimates from the experiment for short-term and longer-term academic outcomes. We first compare the performance of female and male students in Chemistry labs. Using the rescaled (mean = 0 and standard deviation = 1) score in the course, we find that female students perform modestly better than male students. The overall mean and median differences between female and male students are 0.275 and 0.208 standard deviations, respectively. The female median lies at the 59^th^ percentile of the male distribution. If we control for students characteristics including the student’s performance in Chemistry 1A (which is taken prior to the term) we find that female students have a 0.34 standard deviation higher score in the class than male students. female students score 0.358 standard deviations higher on average than male students.

To examine the effects of gender interactions on performance in the Chemistry labs, we estimate several regressions and report the coefficient estimates in Table 1. See Methods section for details on the regression equation, estimation technique, included controls, randomization process, and balance check. Specification 1 reports estimates for the continuous score in the lab course. Female students do no worse when randomly partnered with a male student than when randomly partnered with a female student. We find a coefficient estimate on the gender interaction that is essentially zero and is estimated very precisely. The point estimate is 0.0038 and the 95 percent confidence interval is [-0.0625, 0.0700], which rules out even small negative or positive effects on course scores. Although a large percentage of female college students in STEM report experiencing gender bias from male peers on surveys [10, 11] we do not find evidence that male partners negatively affect their performance. Another interesting finding is that male students are also not affected by having female partners, suggesting that gender interactions are symmetric.

The results are similar when we examine additional measures of performance in Chemistry labs. Specification 2 reports estimates in which the dependent variable is the grade in the course on a 4-point scale, and Specification 3 reports estimates for whether the student passed the course. Using both alternative measures of course performance, female students are unaffected when randomly partnered with male students.

Gender interactions in STEM fields might operate along different channels than course performance. For example, female students might decide to drop Chemistry labs when randomly assigned to a male partner before a score or grade is recorded in the system. Dropping the course could have subsequent consequences such as disrupting the student’s trajectory in the major or even causing the student to leave STEM. Specification 4 reports regression estimates for whether the student dropped the course. We find that female students are not more likely to drop the lab course when they are partnered with a male student.

We also estimate separate regressions using the female and male subsamples. Estimates are reported in the supplementary S2A Table in S1 Appendix for women and S2B Table in S1 Appendix for men. We find similar results. For all of these outcomes, we find no evidence that female students are negatively affected by having male partners relative to female partners.

**Fig 1:**
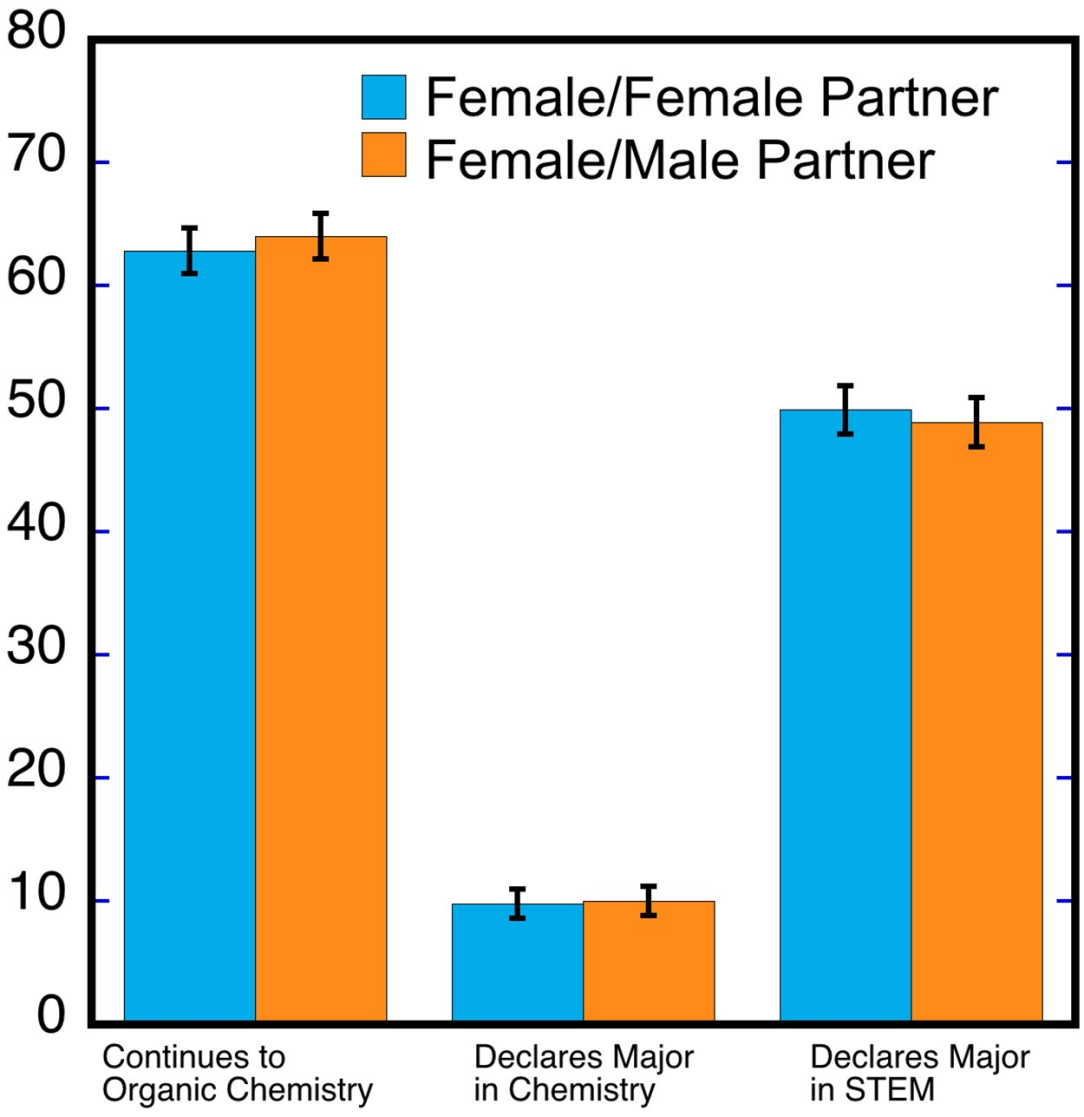
Continued participation in STEM courses among female students as a function of laboratory partner. Beyond laboratory performance, the data were analyzed to assess continued STEM participation by examining enrollment in the more advanced course of organic chemistry, as well as declaration of major. The data show that female students persist in their interest of STEM regardless of lab partner gender. Errors bar reflect 95% confidence interval.

Gender interactions may have longer-term consequences such as dissuading female students from continuing in Chemistry (specifically, to organic chemistry) and STEM. Fig 1 examines whether male partners negatively influence subsequent course taking and majoring in Chemistry and STEM by female students (see also S3 Table in S1 Appendix). In these regressions and for the subsample of students taking both labs, we implicitly treat the experimental intervention as taking on one of three values for each female student for having a male partner (i.e. a 0, 0.5 or 1). For all of these measures of longer-term interest in continuing in STEM, we do not find evidence that female students are negatively affected when partnered with male students. Gender interactions with partners in the labs do not cause women to leave STEM, which is consistent with the finding that male partners do not negatively affect grades in the labs or increase the likelihood of dropping the lab course. Using the subsample that takes both lab courses we also estimate specifications for the three longer-term outcomes that includes separate dummy variables for having male partners in both and having a male and female partner. For all three longer-term outcomes, we do not find that either combination of partners by gender has affects. We also estimate the model only using the first Chemistry lab taken by each student. We find similar null results with very small and statistically insignificant coefficients, and less precision as expected.

Chemistry 1A is the first course in the Introduction to Chemistry sequence and is taken before students enroll in the labs. Female students who do well in this course might react differently to male lab partners. For example, receiving a good grade in Chemistry 1A might reduce the likelihood of shying away from competition because they already demonstrated to themselves that that they can do well in a competitive environment. Chemistry 1A is a difficult and competitive course. Grades are factored into whether students are accepted into the selective Chemistry major. In Chemistry 1A, 13 percent of students receive Fs and another 6 percent receive Ds. Only 23 percent of the class receives grades of A- or higher. On the other hand obtaining a lower grade in Chemistry 1A might magnify these concerns and increase dissatisfaction in STEM [7]. Using grades in Chemistry 1A we estimate the effects of male partners separately for low and high ability female students. High ability is defined as receiving a B grade or higher in Chemistry 1A (which represents the top 46 percent of the distribution) and low ability is defined as receiving a B- grade or lower. The results are not sensitive to when we used alternative cutoffs such as B+ or higher (32 percent of the distribution) or B- or higher (56 percent of the distribution) to define the higher-ability group. Although, on average, female students do not appear to be affected by being partnered with male students there could be offsetting negative and positive effects for female students based on their performance in Chemistry 1A.

Table 2 reports regression estimates for the same four course outcomes as reported in Table 1 but the specifications now include interaction terms for low and high ability students based on performance in Chemistry 1A. We find that both low and high ability female students are not affected by male partners in Chemistry labs. We also find symmetrically that there is no evidence suggesting that low or high ability male students are affected when partnered with female students relative to being partnered with male students.

The ability level of the partner might also affect gender interactions in Chemistry labs. If female students are partnered with male students of high ability then it might increase the potential for gender bias or fear of competition. On the other hand, female students who are partnered with low ability male students might not face these negative influences or, alternatively, may perform “down” to the level of the low ability male partner. The null effect estimated above might simply represent offsetting negative and positive effects for the two different situations. We examine separate estimates of partner gender effects by whether the partner was high ability (i.e. B or higher in Chemistry 1A) or low ability (i.e. B- or lower in Chemistry 1A). The regressions specification is Eq (2). Table 4 reports estimates for the four course outcome measures but now includes interaction terms with low and high ability partners. We find no evidence that the partner’s ability level matters. Female students are not affected when paired with a low ability male student or when paired with a high ability male student. Similarly, male students are unaffected by the ability level of female partners.

**Table 4:** Regression coefficients for main outcomes by ability of partner. Linear regressions control for baseline lab sections, full grade distribution in Chem 1A prior to labs, ethnicity, gender, Educational Opportunity Programs status, year in college, major interest, and declaration of major. Standard errors (in parentheses) are clustered by lab sections.

		Numeric score	Grade (4 point scale)	Passed course	Dropped course
		1	2	3	4
**Female student with**					
	low ability male partner	0.0367	-0.0036	-0.0009	0.0031
		(0.0436)	(0.0203)	(0.0112)	(0.0113)
	high ability male partner	-0.0164	-0.0139	0.0091	-0.0097
		(0.0388)	(0.0175)	(0.0098)	(0.0099)
**Male student with**					
	low ability female partner	0.0068	-0.0128	0.0040	-0.0005
		(0.0645)	(0.0300)	(0.0124)	(0.0118)
	high ability female partner	0.0627	0.0174	0.0112	-0.0070
		(0.0563)	(0.0263)	(0.0125)	(0.0121)
Observations		4,963	4,971	5,241	5,241
R-squared		0.2846	0.1808	0.1075	0.106
Mean (Dep. var.)		0.0005	3.8219	0.9475	0.0500
SD (Dep. var.)		0.9998	0.4439	0.2230	0.2179

*** p<0.01, ** p<0.05, * p<0.1.

The importance of role models in education has been well documented [6, 19–21]. Over the course of the experiment, there were 330 unique lab sections run by 71 different PhD student teaching assistants of which 45 percent were women. The presence of a female role model as a PhD student teaching assistant might offset any potential negative effects of male partners on female students. Furthermore, having a female teaching assistant could reduce discrimination against female students by altering the atmosphere of the lab.

Table 5 reports regression estimates of male partner effects based on the gender of the teaching assistant assigned to the section. Teaching assistant assignments across labs are not known to students prior to online enrollment. We find that in labs with either a female or male teaching assistant, having a randomly assigned male partner has no effect (negative or positive) on female students. In environments with and without TA role models and different potential levels of gender bias we find no evidence that female students are affected by male lab partners.

**Table 5:** Regression coefficients for main outcomes by gender of the teaching assistant. Linear regressions control for baseline lab sections, full grade distribution in Chem 1A prior to labs, ethnicity, gender, gender by TA gender, Educational Opportunity Programs status, year in college, major interest, and declaration of major. Standard errors (in parentheses) are clustered by lab sections.

		Numeric score	Grade (4 point scale)	Passed course	Dropped course
		1	2	3	4
**Female student partnered with a male in a lab section with a**					
	female TA	-0.0066	-0.0162	0.0012	-0.0010
		(0.0556)	(0.0255)	(0.0117)	(0.0116)
	male TA	0.0143	-0.0036	0.0081	-0.0074
		(0.0414)	(0.0188)	(0.0119)	(0.0118)
**Male student partnered with a female in a lab section with a**					
	female TA	0.0391	0.0066	0.0326**	-0.0284**
		(0.0771)	(0.0341)	(0.0151)	(0.0142)
	male TA	0.0346	0.0009	-0.0146	0.0182
		(0.0709)	(0.0327)	(0.0146)	(0.0141)
Observations		4,936	4,944	5,212	5,212
R-squared		0.2833	0.1807	0.1094	0.1078
Mean (Dep. var.)		-0.0039	3.8211	0.9476	0.0499
SD (Dep. var.)		1.0010	0.4448	0.2228	0.2177

*** p<0.01,

** p<0.05,

* p<0.1.

## Discussion

Using a novel, large-scale experiment that randomly pairs female and male students in introductory Chemistry labs at a large public research university, we explore gender interactions in STEM. The findings are surprising. Although students work one-on-one the entire term in Chemistry labs and despite previous reports of gender bias, the findings indicate that female students are not negatively affected academically by male partners. Female students do no worse when paired with male students than when paired with female students. Female students do not receive lower scores or grades, and they are no more likely to drop the course or not continue in Chemistry or another STEM field. These findings on gender interactions in the labs are likely generalizable and not related to any specific set of scientific concepts or skills given the broad range of capabilities required in introductory chemistry laboratories.

Although previous studies provide evidence that female students self-report experiencing gender bias in STEM fields from classmates [10, 11, 12], we find no evidence of negative effects from male classmates on key academic outcomes such as course grades and pass rates, and continuation in Chemistry and STEM. Gender bias and discrimination might indeed exist, but in one of the most intensive interactions between classmates possible (i.e. pairwise interactions in labs for an entire term instead of interactions in a larger lecture-based classroom) female students are not doing worse academically when randomly paired with male students.

Diving deeper into additional interactions with student ability, we find no evidence suggesting that academically weaker female students are negatively affected by male students and no evidence that female students are negatively affected when paired with academically stronger male students (both of which might suggest strong gender bias and competition threats to female students). The presence of a female role model, represented by a PhD student teaching assistant running the lab, also does not alter the effects of the interaction with male students.

Although significant concerns continue over the underrepresentation of women in STEM fields, our findings suggest that female students are not being dissuaded from Chemistry by male peers in the early stages of their academic careers. One fruitful future direction for increasing female participation in STEM might be to focus on expanding interest in STEM by emphasizing the salience of jobs in these fields for solving broader world problems [2, 9, 22]. Even so, systemic change will require strong and continuing support at all levels of education by university leaders, policymakers and others [23, 24]. But the stakes are high and worth the investment as increasing women in STEM is likely to reduce earnings inequality and represents a vast resource for economic growth in countries where female labor participation has been historically low [25]. One recent study of undergraduates found that high-ability women give up as much as $13,000–$20,000 in annual salary by choosing non-STEM majors [26]. Future research should investigate gender interactions in latter STEM courses, but the positive results found here are promising for the future prospects of women in STEM fields.

## Supporting information

S1 Appendix (PDF)
