# Supplementary material for: The effects of male peers on the educational outcomes of female college students in STEM: Experimental evidence from partnerships in Chemistry courses
Source: PLoS One. 2020 Jul 9;15(7):e0235383. doi: 10.1371/journal.pone.0235383 (PMC7347198; doi:10.1371/journal.pone.0235383)
Supplement: S1 Appendix — (PDF) [file pone.0235383.s001.pdf]

A New Generation of Female Scientists? A Quantitative Assessment of Interactions between Female and Male Students in Gateway Chemistry Labs

Supplementary Material

December 16, 2019

Robert Fairlie, Glenn Millhauser, Daniel Oliver, Randa Roland
Correspondence to: rfairlie@ucsc.edu and glennm@ucsc.edu

This PDF file includes:
Tables S1 to S6

Additional Tables

Table S1 reports a comparison of student characteristics for lab students, all students at the university, students receiving Chemistry degrees, and U.S. students receiving Chemistry and STEM degrees.

Table S1: Comparisons of Lab Students to Other Groups of Students

	Female Lab Students	All Lab Students	UCSC Female Students	UCSC All Students	UCSC Majors in Chemistry	U.S. Bachlor Degrees in Chemistry	U.S. Bachlor Degrees in STEM	U.S. Bachlor Degrees in All Fields
Female		58.2%		48.9%	43.0%	48.2%	35.1%	57.1%
White	27.6%	29.5%	29.8%	30.3%	42.3%	58.0%	61.9%	63.9%
Asian	30.8%	31.4%	25.3%	27.9%	31.3%	13.5%	12.1%	7.1%
Hispanic/Latinx	30.0%	27.3%	31.6%	26.7%	35.0%	9.7%	9.6%	11.5%
African-American	2.5%	2.1%	5.2%	4.3%	1.7%	6.9%	6.7%	10.1%
EOP	37.1%	34.2%		34.7%				
Freshman	25.9%	24.6%	21.8%	22.4%				
Sophomore	60.0%	58.9%	20.8%	20.2%				
Junior	10.9%	12.3%	27.5%	28.3%				
Senior	3.2%	4.3%	29.9%	29.1%				

Notes: 1) The reported columns for UCSC students are from published reports from Institutional Research, Assessment and Policy Studies for various years. 2) U.S. Degree data are from the National Center for Educational Statistics Digest of Educational Statistics (2014-15 data) and NSF NCSES Data (2015).

Table S2A examines whether the main results for course outcomes are robust to using only the female subsample. The Table reports the same outcomes and includes the same controls at Table 2 reported in the main paper. The estimates are similar and continue to indicate that female students are not affected by whether their partner is male or female.

Table S2A: Regression coefficients for main outcomes from the female sub-sample.
Linear regressions control for baseline lab sections, full grade distribution in Chem 1A prior to labs, ethnicity, Educational Opportunity Programs status, year in college, major interest, and declaration of major. Standard errors (in parentheses) are clustered by lab sections. *** p<0.01, ** p<0.05, * p<0.1.

	Numeric score	Grade (4 point scale)	Passed course	Dropped course
	1	2	3	4
Female student with	-0.0069	-0.015	0.0084	-0.0088
male partner	-0.0357	-0.0159	-0.0088	-0.0088
Observations	2,888	2,895	3,045	3,045
R-squared	0.3392	0.2015	0.1488	0.1487
Mean (Dep. var.)	0.1148	3.8689	0.9514	0.0483
SD (Dep. var.)	0.8461	0.3511	0.2151	0.2144

Table S2B examines whether the main results for course outcomes are robust to using only the male subsample. The Table reports the same outcomes and includes the same controls at Table 2 reported in the main paper. The estimates are similar and continue to indicate that male students are not affected by whether their partner is female or male.

Table S2B: Regression coefficients for main outcomes from the male sub-sample.
Linear regressions control for baseline lab sections, full grade distribution in Chem 1A prior to labs, ethnicity, Educational Opportunity Programs status, year in college, major interest, and declaration of major. Standard errors (in parentheses) are clustered by lab sections. *** p<0.01, ** p<0.05, * p<0.1.

	Numeric score	Grade (4 point scale)	Passed course	Dropped course
	1	2	3	4
Male student with	0.0256	0.0017	0.0071	-0.0035
female partner	-0.0636	-0.029	-0.0125	-0.0117
Observations	2,080	2,081	2,201	2,201
R-squared	0.3198	0.2601	0.197	0.203
Mean (Dep. var.)	-0.1593	3.7565	0.9423	0.0522
SD (Dep. var.)	1.1625	0.5406	0.2332	0.2226

Table S3 examines whether male partners affect female student interest in continuing in Chemistry or STEM more generally. Estimates of Equation (1) are reported for four additional outcomes. First, we estimate whether male partners negatively influence subsequent course taking in Chemistry and STEM courses by female students. Specification (1) reports estimates of gender interactions when the dependent variable is future enrollment in the secondary sequence in organic chemistry. Specification (2) switches the focus to declaring majors in Chemistry, and Specification (3) explores declaring majors in any STEM field. Specification (4) explores the impacts on grades in the large lecture Chemistry courses taken concurrently with the lab. For all of these measures of longer-term interest in continuing in STEM, we do not find evidence that female students are negatively affected when partnered with male students.

Table S3: Regression coefficients for secondary outcomes. Linear regressions control for baseline lab sections, full grade distribution in Chem 1A prior to labs, ethnicity, gender, Educational Opportunity Programs status, year in college, major interest, and declaration of major. Students that have declared as a STEM major prior to enrolling in the lab are excluded from columns (2) and (3). Standard errors (in parentheses) are clustered by lab sections. *** p<0.01, ** p<0.05, * p<0.1.

	Takes Organic Chemistry	Declared Chemistry	Declared STEM	Co-current lecture grade
	1	2	3	4
Female student with male partner	0.0115	0.0026	-0.0100	-0.0050
	(0.0165)	(0.0104)	(0.0198)	(0.0319)
Male student with female partner	-0.0165	0.0117	-0.0060	0.0360
	(0.0193)	(0.0146)	(0.0224)	(0.0368)
Observations	5246	4,878	4,878	4,357
R-squared	0.295	0.2525	0.2062	0.3762
Mean (Dep. var.)	0.6281	0.0976	0.4992	2.9248
SD (Dep. var.)	0.4834	0.2968	0.5001	0.8533

Table S4 examines the sensitivity of the main results to the chosen definition of student's ability. As a robustness check, we distinguish between low and high ability based on a student's performance in all previous courses (i.e. prior GPA). We use the median prior GPA for all students to define the low vs. high ability cutoff which is 3.21. Table S3 shows that using this measure we find that low-ability female students are not affected by male partners in Chemistry labs. High ability female students are also not affected by male partners.

Table S4: Regression coefficients for main outcomes by ability (using pre-GPA). Linear regressions control for baseline lab sections, prior GPA, ethnicity, gender, gender by ability, Educational Opportunity Programs status, year in college, major interest, and declaration of major. Standard errors (in parentheses) are clustered by lab sections. *** p<0.01, ** p<0.05, * p<0.1.

	Numeric score	Grade (4 point scale)	Passed course	Dropped course
	1	2	3	4
Female student of low ability with male partner	0.0207	-0.0041	-0.0040	0.0043
	(0.0509)	(0.0260)	(0.0133)	(0.0134)
Female student of high ability with male partner	-0.0188	-0.0137	0.0122	-0.0111
	(0.0367)	(0.0148)	(0.0109)	(0.0109)
Male student of low ability with female partner	0.0104	-0.0130	0.0180	-0.0109
	(0.0691)	(0.0335)	(0.0138)	(0.0131)
Male student of high ability with female partner	0.0553	0.0123	0.0033	-0.0025
	(0.0484)	(0.0213)	(0.0124)	(0.0120)
Observations	4,920	4,928	5,192	5,192
R-squared	0.3477	0.2196	0.1045	0.102
Mean (Dep. var.)	0.0022	3.8229	0.9482	0.0493
SD (Dep. var.)	0.9981	0.4426	0.2217	0.2165

Table S5 examines the sensitivity of the main results to the chosen definition of partner's ability. As a robustness check, we distinguish between low and high ability based on the partner's performance in all previous courses (i.e. prior GPA). Table S4 shows that using prior GPA to define low and high ability partners does not change the results. Female students are not negatively affected by either high ability male partners or low ability male partners.

Table S5: Regression coefficients for main outcomes by ability of partner (using pre-GPA).
Linear regressions control for baseline lab sections, prior GPA, ethnicity, gender, Educational Opportunity Programs status, year in college, major interest, and declaration of major. Standard errors (in parentheses) are clustered by lab sections. *** p<0.01, ** p<0.05, * p<0.1.

	Numeric score	Grade (4 point scale)	Passed course	Dropped course
	1	2	3	4
**Female student with**				
low ability male partner	-0.0141	-0.0143	0.0033	-0.0022
	(0.0379)	(0.0176)	(0.0105)	(0.0106)
high ability male partner	0.0241	-0.0009	0.0065	-0.0062
	(0.0400)	(0.0189)	(0.0112)	(0.0114)
**Male student with**				
low ability female partner	0.0080	-0.0126	0.0009	0.0022
	(0.0597)	(0.0282)	(0.0127)	(0.0119)
high ability female partner	0.0645	0.0170	0.0166	-0.0120
	(0.0519)	(0.0265)	(0.0121)	(0.0118)
Observations	4,968	4,976	5,246	5,246
R-squared	0.3425	0.215	0.1044	0.1022
Mean (Dep. var.)	0.0000	3.8219	0.9476	0.0499
SD (Dep. var.)	1.0000	0.4438	0.2229	0.2178

Table S6 explores whether the share of female students in the lab interacts with having a male partner. If gender bias is stronger in STEM classes with a relatively high percentage of male students then we might find negative effects of being partnered with males in those classes. Table S5 reports regression estimates for the four course outcome measures including interaction terms for high (majority) or low (non-majority) female shares of students in the lab. We find that having a male partner in labs with either high or low female shares has no effect on female students. For male students, we also find no interaction effect with the share of female students in the lab.

Table S6: Regression coefficients for main outcomes by majority female lab sections. Linear regressions control for baseline lab sections, full grade distribution in Chem 1A prior to labs, ethnicity, gender, gender by majority gender of lab section, Educational Opportunity Programs status, year in college, major interest, and declaration of major. Standard errors (in parentheses) are clustered by lab sections. *** p<0.01, ** p<0.05, * p<0.1.

	Numeric score	Grade (4 point scale)	Passed course	Dropped course
	1	2	3	4
**Female student partnered with a male in a**				
majority female lab section	-0.0059	-0.0150	0.0020	-0.0019
	(0.0389)	(0.0185)	(0.0092)	(0.0091)
non-majority female lab section	0.0132	-0.0020	0.0160	-0.0151
	(0.0681)	(0.0279)	(0.0183)	(0.0183)
**Male student partnerned with a female in a**				
majority female lab section	0.0043	-0.0122	-0.0043	0.0071
	(0.0838)	(0.0360)	(0.0168)	(0.0155)
non-majority female lab section	0.0502	0.0112	0.0197	-0.0155
	(0.0674)	(0.0317)	(0.0141)	(0.0138)
Observations	4,968	4,976	5,246	5,246
R-squared	0.2846	0.1808	0.1078	0.1063
Mean (Dep. var.)	0.0000	3.8219	0.9476	0.0499
SD (Dep. var.)	1.0000	0.4438	0.2229	0.2178
